# Effectiveness of Yoga Lifestyle on Lipid Metabolism in a Vulnerable Population—A Community Based Multicenter Randomized Controlled Trial

**DOI:** 10.3390/medicines8070037

**Published:** 2021-07-13

**Authors:** Raghuram Nagarathna, Saurabh Kumar, Akshay Anand, Ishwara N. Acharya, Amit Kumar Singh, Suchitra S. Patil, Ramesh H Latha, Purnima Datey, Hongasandra Ramarao Nagendra

**Affiliations:** 1 Swami Vivekananda Yoga Anusandhana Samsthana (S-VYASA), Bengaluru 560105, India; dramits90@gmail.com (A.K.S.); ayursuch@gmail.com (S.S.P.); chancellor@svyasa.edu.in (H.R.N.); 2 Neuroscience Research Lab, Department of Neurology, Postgraduate Institute of Medical Education and Research (PGIMER), Chandigarh 160012, India; Saurabh.kum1991@gmail.com; 3 Centre for Mind Body Medicine, PGIMER, Chandigarh 160012, India; 4 Centre for Cognitive Science and Phenomenology, Panjab University, Chandigarh 160014, India; 5 Central Council for Research in Yoga & Naturopathy (CCRYN), Delhi 110058, India; acharyaishwar@gmail.com; 6 Yoga Clinic, Bhopal 462026, India; latavk123@gmail.com; 7 Arogya Rasahara Kendra, Bhopal 462024, India; purnimadatey@gmail.com

**Keywords:** diabetes yoga protocol, diabetes, prediabetes, dyslipidemia, lipid regulation

## Abstract

**Background:** Dyslipidemia poses a high risk for cardiovascular disease and stroke in Type 2 diabetes (T2DM). There are no studies on the impact of a validated integrated yoga lifestyle protocol on lipid profiles in a high-risk diabetes population. **Methods:** Here, we report the results of lipid profile values of 11,254 (yoga 5932 and control 5322) adults (20–70 years) of both genders with high risk (≥60 on Indian diabetes risk score) for diabetes from a nationwide rural and urban community-based two group (yoga and conventional management) cluster randomized controlled trial. The yoga group practiced a validated integrated yoga lifestyle protocol (DYP) in nine day camps followed by daily one-hour practice. Biochemical profiling included glycated hemoglobin and lipid profiles before and after three months. **Results:** There was a significant difference between groups (*p* < 0.001 ANCOVA) with improved serum total cholesterol, triglycerides, low-density lipoprotein, and high-density lipoprotein in the yoga group compared to the control group. Further, the regulatory effect of yoga was noted with a significant decrease or increase in those with high or low values of lipids, respectively, with marginal or no change in those within the normal range. **Conclusion:** Yoga lifestyle improves and regulates (lowered if high, increased if low) the blood lipid levels in both genders of prediabetic and diabetic individuals in both rural and urban Indian communities.

## 1. Introduction

Dyslipidemia (altered blood lipids) is as a contributing risk factor for various macrovascular complications in type-2 diabetes mellitus (T2DM) patients [1]. Dyslipidemia is characterized by high levels of triglycerides (≥150 mg/dL), high low-density lipoprotein (LDL ≥ 130 mg/dL), low high-density lipoprotein (HDL < 40 mg/dL for men; <50 mg/dL for women) [2] and high levels of total cholesterol (≥200 mg/dL) [3,4]. It is difficult to define the cut-off range for dyslipidemia, as it varies from study to study due to the difference in methodologies used. Studies suggest that Indian and migrant Asian Indians tend to show increased triglycerides and decreased HDL serum levels than western residents [5]. In comparison, serum cholesterol levels tend to be similar to the US population and lower than the UK population [5]. A high volume of blood cholesterol is associated with greater chances of developing cardiovascular disease, including stroke, peripheral vascular disease, and coronary heart disease (CHD) [6]. This is a major cause of cardiac morbidity and mortality, especially in the aged and in patients with T2DM [6]. According to the American Diabetes Association (ADA), T2DM is associated with a two- to four-fold increased risk of developing CHD and increased triglycerides. Decreased HDL levels are common in patients with T2DM [7,8]. The Indian Council of Medical Research study revealed a high prevalence of dyslipidemia in India, with 79% of the studied population showing an abnormality in at least one lipid parameter [9]. The study found a higher prevalence of abnormal lipids in females than in males. The middle-aged group population (35–64 yrs) showed higher lipid abnormalities than the younger group (20–24 yrs) [9].

The increasing burden of heart disease and T2DM [10], despite billions of dollars spent on research and the use of lipid-lowering drugs over the years, has posed a big challenge for health expenditure [11], and there is an urgent need to investigate cost-effective alternative approaches. Yoga is one of the popular mind-body approaches developed in India [12]. Yoga is known to exert positive physiological changes, which have wide-ranging scientific significance [13,14] as research findings have described the benefits of yoga in managing stress, anxiety, and negative sentiments [12,15]. Yoga may exert cardiovascular changes by acting on neurological pathways like the autonomic nervous system (ANS), sympatho-adrenal medullary (SAM), or hypothalamic pituitary adrenal (HPA) [16]. It is also believed that yoga postures like pranayama (breathing) and asanas improve cardiovascular and respiratory activity by increasing nitric oxide (NO) and antioxidant levels in the blood. Further, the HPA/SAM are hypothesized to reduce the over-production or activation of catecholamines, corticosteroids (glucocorticoids), and subsequent cytokines that are pro-inflammatory, increasing CHD risk [16,17]. β-cell sensitivity in response to glucose metabolism and insulin secretion is improved by these yoga postures [18].

Hypercholesterolemia, hypertriglyceridemia, and hyperlipidemia are significant risk contributors for coronary heart disease. Both prevention and control of coronary heart disease with its associated diseases are essential and can be achieved by modifying the lipid profile [19]. There are many reports on the adverse effects of an increased volume of bad cholesterol (LDL, triglycerides) and reduced volume of good cholesterol (HDL) and on drugs to reduce LDL and increase HDL levels. Some studies have shown the detrimental effects (increased mortality in coronary heart disease) of lowering the levels of cholesterol (<160 mg/dL), which calls for the integration of an effective evidence-based non-pharmacological approach (Cir and EHJ) [20,21]. Mahajan et al. conducted a yoga-based study on the lipid profiles of subjects with coronary artery disease and reported the effectiveness of yoga in risk modification [22]. Another study reported yoga exerted its therapeutic potential in subjects with mild-to-moderate hypertension by reducing the risk of cardiovascular diseases [23]. A systemic review conducted by Innes et al. evaluated the effects of yoga-based controlled trials and found yoga to be effective in managing the blood lipids along with glycemic control [24]. Similarly, Raveendran et al. [25] suggested that daily yoga practice helps maintain overall body growth [25]. Hence, it appears that maintaining the lipid levels within the normal range is essential [26], which can be achieved by integrating yoga with usual care.

There are a few studies on lipid profiles in T2DM patients [27,28], but there are none that have examined the normalizing effect of yoga on lipid values. This requires a large sample size from diverse community cohorts, which is challenging. Hence, the present paper planned to look at the lipid normalizing effect of the yoga lifestyle change program in a trial that was designed as a nationwide multicenter two-armed control trial for primary and secondary prevention of diabetes named *Niyantrita Madhumeha Bharata Abhiyaan* (NMB-2017). The sampled population is vulnerable (high risk) to diabetes with scores ≥60 on the Indian Diabetes Risk Score (IDRS).

## 2. Materials and Methods

### 2.1. Sample Size Calculation

The sample size for the trial was calculated for the primary prevention of diabetes, which was the primary objective of the study. The details of the sample size calculation are provided in our earlier publication [29]. In brief, we used the values of relative risk-reduction after lifestyle intervention in prediabetes subjects, as observed in an earlier study [30], which had an annual conversion rate of 11.1% in the control and 7.8% in the intervention group; based on this we obtained a total sample size of 5320, i.e., 2660/group at *α* = 0.05 and 1 − *β* = 0.80.

### 2.2. Screening and Recruitment

**Figure 1:**
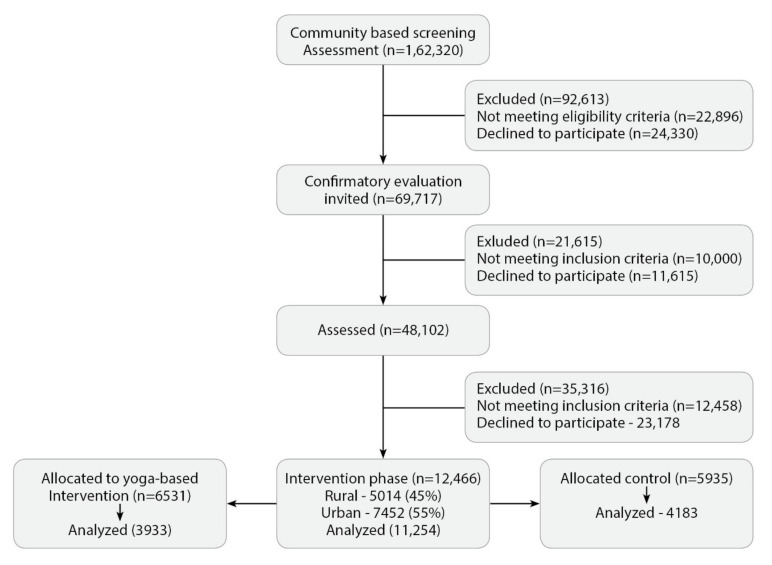
CONSORT diagram of the study.

The screening was carried out after getting permission from the Institutional Ethics Committee of the Indian Yoga Association (IYA) (Reference no: RES/IEC-IYA/001) and obtaining the signed informed consent by all participants. Details of the methodology are communicated in an earlier publication [31]. This was multi-level randomization starting from the randomization of districts, towns, and census enumeration blocks (urban) and villages (rural) depicted in the map (Figure 1, Figure S1) [31]. The study was a cluster randomization design in order to overcome the barriers of contamination. For this reason, we included the entire block or village for yoga intervention or control as the case may be. The yoga and control clusters were separated by 5–10 km. We randomly identified the group as an intervention group, i.e., 2 out of 4 villages and 1 or 2 out of 2 or 4 census enumeration blocks (CEBs) were identified in the selected ward, while the other was assigned as the waitlisted control group. Phase 2 included administering the yoga lifestyle protocol for diabetes developed by an expert committee through the Delphi method in the randomly allocated clusters.

### 2.3. Randomization and Allocation Concealment

Details of randomization have been published in our earlier publication [29]. In brief, a 4-stage randomization approach was implemented using a multi-level stratified cluster sampling method. The study was planned to be in two phases. Phase 1 involved a cross-sectional survey from the entire country using the National Family Health-3 (NFH-3) sampling process. The twenty-nine (95% of India’s population from 2011 census) most populous states/union territories of India were grouped under seven geographical zones (Northwest, North, Northeast, West, Central, East, and South) based on their cultural similarities (Figure S1); random selection process was applied for selecting 65 districts, four rural villages and two census enumeration blocks in an urban town. In brief, this was a trial on randomly selected rural and urban community clusters. Phase 1 was door-to-door screening for IDRS in these randomly selected clusters of rural (villages) and urban (CEBs) locations, followed by blood tests in high-risk (≥60 on IDRS) individuals.

### 2.4. Selection Criteria for Phase 2 (RCT)

In phase 2, two villages and one CEB were randomly selected for yoga intervention, and two villages and one CEB formed waitlist control. Adults (20 years) of both genders with IDRS Score ≥60 and those with known diabetes (any score on IDRS) were included. Those with reported psychiatric problems, major diabetes complications (nephropathy, retinopathy, coronary artery disease, history of cerebrovascular accidents) were excluded. Pregnant women, lactating mothers, and those who had any surgery within 12 months were also excluded. Information about medication was taken. Individuals taking drugs were excluded from the study. The lipid values of those who had reported that they had practiced yoga regularly within the last three months before the camp were not included in this analysis.

### 2.5. Blinding and Masking

Since it was a community-based interventional cluster-randomized trial, the participants, instructors, and other individuals involved in the study were not blinded. Masking was ensured at different levels. The researchers in the central office provided the names of the randomly selected names of clusters to the field Senior Research Fellows (SRFs), and hence the selection bias was avoided. The central laboratory that carried out the blood investigations on the coded samples, the data operator who checked the accuracy of the data obtained online from the field researchers, and the statistician who analyzed the data were blinded.

### 2.6. Assessments

Zonal coordinators, yoga volunteers, and SRFs visited the campsites for formal interaction with the leaders of the towns/villages and conducted door-to-door screening using the IDRS parameters. Those eligible based on the inclusion/exclusion criteria were invited to the blood camp. Two phlebotomists drew the blood samples in coded label vacutainers. The sample was processed for plasma separation, stored and transported in cold containers to the nearest laboratory, and was processed for biochemical analysis within 6 h of blood withdrawal. Data was collected by SRL labs. For storing, the sample standard method was applied. These estimations were carried out by the same NABL accredited laboratory (SRL) on fasting blood samples collected on day one and after three months of the trial in both yoga and control groups. Serum from the fasting venous blood sample was used for the estimation of lipids and glycated hemoglobin (A1c) on an auto-analyzer (Beckman Coulter-Auto-analyzer model 2700/480); the cholesterol esterase oxidase-peroxidase-amidopyrine method for TC, the glycerol phosphate oxidase-peroxidase-amidopyrine method for TG and polyethylene glycol-pretreated enzymes for HDL [29,31]. The criteria for diabetes and prediabetes were based on A1c values. ADA guidelines recommend A1c based screening as the most practical measure to segregate the population into healthy (A1c < 5.3%), prediabetes (A1c: 5.3–6.4%), and diabetes (A1c ≥ 6.5%) considering the impending practical challenges in screening a large population.

### 2.7. Quality Assurance and Training

Each zone had a zonal coordinator, 35 SRFs (approximately 1 per 2 districts), 1200 certified yoga volunteers, and 2 research associates. The zonal training program organized in different zones trained the SRFs in organizing the camps, data acquisition, volunteer training, using the mobile app, maintaining logbooks, conducting regular meetings, etc. These SRFs further trained the volunteers in their respective areas. We used both paper and mobile apps to capture the data, collated by deep learning and big data analysis.

### 2.8. Intervention

Both groups were given the standard medical advice on lifestyle for the prevention and management of diabetes under the doctor’s supervision from the local medical center. The advice on lifestyle through detailed interactive group lectures included (a) advice on a healthy diet for diabetes; (b) regular and timely exercise (walking for more than 20 min daily); (c) habits (sleep, hygiene, tobacco, alcohol, mobile addiction etc.); and (d) stress management.

#### Diabetes Yoga Protocol

The yoga group received a validated diabetes yoga lifestyle protocol (DYP) developed by an expert committee of 16 professionals (yoga masters, yoga researchers, and diabetologist) with two rounds of interaction using the Delphi technique with a CVR > 0.7. The reliability of the protocol was tested by cluster analysis with an interclass coefficient value of 0.05. The details of the expert committee are published earlier [29].

The 60-min yoga protocol (Table S1) for prediabetes and uncomplicated diabetes consisted of yoga postures, breathing practices, relaxation, pranayama, meditation, and lectures on yogic lifestyle for behavioral modification (diet, sleep, stress management through conceptual correction using jnana yoga, bhakti yoga and karma yoga). During the initial 9-day introductory camps, daily feedback for any adverse effect was recorded. After this period, they were asked to do the practices at home using handbooks and/or DVDs; adverse effects were discussed and documented during the weekly follow-up classes of 2 h for three months. Fidelity data was documented through the attendance sheet and regular follow-up calls on the phone; individual pictures and videos were also recorded and archived in a retrievable format. No financial incentive was given to the participants. Any adaptation was decided locally by the trained instructors, which was done based on individual cases and not for the area as a whole. The standard protocol was implemented in all places uniformly by trained, certified instructors, e.g., some patients who could not squat on the floor for physical postures were taught a modified version of the postures to be done sitting in a chair. This was included and taught to the instructors in their 5-day orientation programs (5 days each in 20 orientation camps in different zones) [31].

### 2.9. Statistical Analysis

Data were analyzed using SPSS version 21.0. The matching of data from different sources and different time points was checked by fuzzy logic. Independent samples *t*-test was used to compare baseline characteristics of the two groups. Paired samples *t*-test was used for pre-post comparison within groups. Pre-post comparisons between yoga and control groups were checked by the ANCOVA test. The difference in deference analysis was done by multinomial regression. A mixed linear model was used to check the differences between subcategories of lipid values in 3 subgroups of A1c.

## 3. Results

Figure 1 shows the consort diagram. In the first phase of pan-India screening from seven zones (Figure S1), data on IDRS and known diabetes were available on 162,330 individuals from the randomized urban (52%) and rural (48%) clusters. Of these, 69,717 individuals at high risk and who had known diabetes were invited for detailed assessments, and 48,102 responded. Based on A1c values, 6094 were found to be newly diabetic (A1c ≥ 6.5), 7920 were in the prediabetes range, and 13,597 were in the normoglycemia range and were invited for intervention in phase 2 of the trial. Of the 12,466 who participated in the trial, 6531 were in the yoga clusters and 5935 in the control clusters from all zones. The main reason for non-response, although they were interested in participation, was time constraints due to family or occupational commitments. Of these, follow-up data were available on 11,254 (5932 yoga and 5322 control; 9% drop out) at three months; analysis of pre-post lipid profile data was done on 8116 (3933 yoga and 4183 control) individuals after excluding extreme values.

The baseline characteristics (Table 1) revealed a non-significant difference between groups in the age and gender distribution (independent samples *t*-test) between the yoga and control groups. The mean (SD) age (years) of the yoga and control groups was 48.70 ± 10.64 of 48.41 ± 10.22, respectively. In the yoga group, the percentage of the male population was 42.8%, and in the control group, it was 40.9%. In the yoga group, the proportion of the rural population was 31.5%, while in the control, it was 47.3%. There were 4896 subjects in urban (2693 yoga and 2203 control) and 3220 subjects in rural locations (1240 yoga and 1980 control). By profession, most of the recruited participants were clerical or shop owners, followed by skilled workers. A total of 502 (n) participants in the yoga group and 616 (n) in the control group were aware of their diabetes status/that they had diabetes for the last five years. Many participants were newly diagnosed with diabetes (1105 in the yoga group and 2145 in the control group) (Table 1).

The group analysis (Table 2) showed that TC was reduced significantly (*p* < 0.001 paired *t*-test) from 181.80 ± 39.75 mg/dL to 176.64 ± 38.59 mg/dL after yoga, while there was an increase in the control group from 183.44 ± 40.33 mg/dL to 193.27 ± 47.27 mg/dL. A marginal increase (153.51 ± 72.88 mg/dL) in TG was observed in the yoga group after three months, whereas in the control group, the mean value increased from 155.86 ± 79.40 mg/dL to 191.12 ± 107.44 mg/dL. We found a significant reduction in LDL in the yoga group from 103.54 ± 34.09 mg/dL to 98.65 ± 33.67 mg/dL; the control group instead showed an increase in LDL from 103.99 ± 33.00 mg/dL to 108.01 ± 40.4 mg/dL. Blood HDL showed a reduction in both groups; the yoga group reduced marginally from 49.30 ± 11.48 mg/dL to 48.61 ± 11.55 mg/dL; similarly, the control decreased from 48.92 ± 11.53 mg/dL to 44.62 ± 12.15 mg/dL. Similar changes were found in TG, LDL, and HDL (Table 2).

Analysis of covariance (Table 2) between groups showed that there was a significantly better improvement in the yoga than the control group in TC, TG, LDL, and HDL (*p* < 0.001 ANCOVA). There was a significant difference between groups (*p* < 0.001 Mixed Linear Model analysis) in two subgroups (Table 3) of A1c, i.e., in individuals in the diabetic and prediabetic ranges. There was a non-significant difference between the yoga and control groups in the normoglycemic subgroup.

An interesting observation emerged in the yoga group when we looked at the three subcategories of baseline lipid values, i.e., those with less than, more than, or within the normal range (Table 3, Figure S2). In diabetics (A1c ≥ 6.5) with baseline levels of TC above the normal range, there was a significant reduction, and for those below the normal range, there was a significant increase with a non-significant change in those within the normal range. Looking at TG, LDL, and HDL, there was a significant reduction in those within and above normal ranges with an increase in those below the normal range. This phenomenon of a shift towards normalcy was not seen in the control group. Although there was a significant increase in those below normal values, there was a significant increase in those with average and high TC, TG, LDL, and HDL values. Sub-group analyses showed that there were no significant differences between males and females, urban and rural areas, or young (<40 yrs) and old (>40 yrs) age groups (*p* > 0.05 ANCOVA) in any of the lipid variables (Table 4).

## 4. Discussion

### 4.1. Yoga as an Effective Tool

After three months of intervention, there was a noteworthy decline in the blood lipid (TC, TG, LDL) of subjects with diabetes, prediabetes, and non-diabetes. Interestingly, the values in all three groups showed a similar trend with a significant increase in those with low levels and a decrease in high values in the yoga group, e.g., there was a significant increase in those with HDL <45 mg/dL, while the levels decreased in those with high values of ≥60 mg/dL. A similar regulating effect was seen in TC, TG, and LDL, pointing to the regulatory effect of yoga in normalizing the values to reach a healthy status, i.e., increasing if it was low and decreasing if it was high, which has not been reported earlier.

Similar effects were observed in the case of both males and females. After three months of intervention, a reduction in the vital lipid parameters such as TC and LDL was observed for both the subgroups (male and female). However, TG was found to be elevated in both genders. Rural and urban populations differ in some of the basic characteristics such as relative pollution, lifestyle, diet, and stress. It was observed that yoga induced the same level of changes in biochemical markers in urban populations as in rural populations. Age is one of the important contributing risk factors associated with diabetes [32]. We categorized the study population into two groups based on age, i.e., below or above 40 years. Analysis on the basis of this categorization revealed that yoga is as effective in the aged population (>40 yrs) as in the younger population (<40 yrs).

Several studies have shown the positive effects of yoga in T2DM, although none had used a national consensus protocol [33]. A study on the effect of *pranayama* and *yogasanas* on blood glucose and lipid profile in a two-armed design on 60 patients of T2DM between 35–60 yrs with diabetes recruited from the diabetes clinic of a hospital in Delhi, India, had shown a significant reduction in serum insulin, blood glucose (fasting and postprandial), LDL, TG, and VLDL with an increase in HDL, with insignificant changes in the control group after 45 days [34]. A short-term study based on *asanas*, relaxation, and *pranayama* had also shown a reduction in the lipid profile within nine days of intervention [35]. A systematic review of original studies on the metabolic and clinical effects of yoga in adults with T2DM summarized the beneficial effects of several variables, including anthropometric, blood pressure, glucose tolerance, insulin sensitivity, etc. This study concluded that to realign the global focus towards yoga, better quality studies using standardized yoga programs are required to validate the effects in populations with T2DM [24].

The lipid regulating effect of the integrated yoga lifestyle module has been highlighted in this study as noted by the significantly higher number of subjects shifting from high or low values to the normal range. The state of health, as cited in yoga literature, is defined as a state of dynamically changing functioning of the tissues to achieve a balance (*samatvam yoga*) under varying internal (psychological or biochemical) or external (environmental) situations [36]. Yoga masters evolved several techniques to achieve mastery over the mind (*chittavrittinirodhaha*) that monitors lifestyle behavior through mindfulness [37]. Healing during disease is through restoring this (*samatvam)* harmony or homeostasis. Yoga offers several techniques for correcting the imbalance at mental, emotional, and physical levels, which are postulated to manifest in biochemical changes [38]. Several studies on yoga have shown a similar harmonizing effect. For example, an improved autonomic balance was seen in healthy volunteers [39] and those with heightened sympathetic tone [40]. Studies have also shown restoration of diurnal cortisol rhythm in patients [41].

The efficacy of yoga in achieving this metabolic biochemical homeostasis through lipid normalizing effect opens up several research questions about the mechanism of yoga. Future studies are imperative at the cellular level to examine whether yoga improves LDL receptor sensitivity or receptor-mediated endocytosis and receptor recycling based on the feedback regulation of receptors [42]. Whether the regulatory effect of yoga on HDL is mediated through a reverse cholesterol transport mechanism [43] that includes macrophage cholesterol efflux in arteries or an antioxidant or anti-inflammatory effect [44,45,46] mediated by NO promoting activity needs to be evaluated [43]. The increased number of diabetes cases in the Indian population creates a substantial economic burden [47,48,49,50] and poses a threat to having age-related disorders [51,52,53]. Therefore, the study may be extrapolated to other populations based on the varied acceptability of yoga protocol as its perceived benefits, barriers, and compliance may vary from one country to another. However, the generalizability of the yoga protocol to the other parts of the country may also depend on the availability and acceptability of certified yoga practitioners. Besides, it is difficult to predict the sustainability of the intervention for which long-term studies are required, although the benefits experienced by the participants are likely to attract them in long-term practice. With the establishment of 150,000 wellness centers in the country [54], the long-term sustainability and generalizability to other populations of the intervention may also depend on the extent to which it is integrated with modern medicine and prescribed by physicians or public health enthusiasts.

### 4.2. Strengths

This is the first multicenter nationwide study in both rural and urban populations on the effect of yoga lifestyle on lipid levels. Additionally, this intervention involved certified yoga therapists (checked by inter-rater reliability testing) from several member institutions of the IYA. There were no adverse/serious events reported during the intervention period of three months. However, a few incidents of minor events like pain in the knee or spine or generalized body pain, or issues related to digestion were reported. These issues were handled by the yoga instructors by correcting their postures and advising on relaxation or counter postures.

### 4.3. Limitations of the Study

As this was an interventional study, double-blinding was not possible as the instructor and the participant did know that they were doing yoga as a therapy. The post values of lipid profile were not available in all those who participated due to various reasons: (a) dropouts from the study after recruitment because of time constraints; (b) left the residence in the recruited cluster zone for business or change of jobs although they had participated in 80% of the sessions; (c) blood samples were corrupted (hemolyzed) in a few cases; (d) post data could not be collected due to local conditions such as weather (heavy snowfall (Kashmir valley), extremes of temperature (50 °C in Odisha), heavy rainfall (western ghats of Karnataka) etc.).

Although efforts were made to ensure reliability by several levels of supervision to promote punctuality and uniformity of the intervention, we could not ensure perfect monitoring due to weather conditions (Odisha and Kashmir), local political disturbances (election drive in UP), state-level agitation (Manipur), transfer of yoga volunteers (Kerala and Goa), etc.

## 5. Conclusions

The data presented in this study prompts more detailed investigations, including molecular genetics and cell culture approaches, to understand the mechanism of yoga protocols on lipid metabolism in general. The results indicate a non-redundant impact of yoga intervention, calling for its integration in medical institutes, further increasing the scope of convenience sampling through larger interdisciplinary studies on patients with dyslipidemia and diabetes. Further, with the launch of 150,000 wellness centers by the Government of India, these trained instructors could be used to provide daily modules of DYP, assisted by ASHA and multipurpose workers. Their services could also be utilized for screening and control of other non-communicable diseases. In this manner, regular yoga sessions in each district could lead to health promotion, prevention, and management of diabetes. As these centers will represent a triage of modern medicine, AYUSH, and science investigators, such integration could further lead to increased acceptance and incorporation into the NPCDCS in an evidence-based manner through new protocols for cancer palliative care, cardiovascular disease, and stroke prevention.

## Figures and Tables

**Table 1:** Demographic details of yoga and control groups.

Demographic Details			Yoga	Control	*p* Value
Age (years)	Mean ± SD		48.7 ± 10.64	48.41 ± 10.22	0.03
Gender	Male N (%)		1682 (42.8%)	1710 (40.9%)	<0.001
	Female N (%)		2251 (57.2%)	2473 (59.1%)	
Area	Urban N (%)		2693 (69.5%)	2203 (52.7%)	<0.001
	Rural N (%)		1240 (31.5%)	1980 (47.3%)	
Occupation	Profession		594	778	0.05
	Semi-Profession		106	130	
	Clerical, Shop owner		1415	1405	
	Skilled worker		1321	1345	
	Semi-skilled worker		90	95	
	Unskilled worker		153	170	
Diabetes status	Self-declared Known DM	<5 yrs	502	616	<0.001
		5–10 yrs	163	220	
		>10 yrs	160	209	
	Newly diagnosed DM		1105	2145	
	Pre-diabetes		806	678	
	No DM, only high risk on IDRS		1197	315	

**Table 2:** Changes in lipid variables before and after three months in the two groups. *N* = 8116 (Yoga-3933, Control-4183).

Group	TC_Pre (mg/dL)	TC_Post (mg/dL)	TG_Pre (mg/dL)	TG_Post (mg/dL)	LDL_Pre (mg/dL)	LDL_Post (mg/dL)	HDL_Pre (mg/dL)	HDL_Post (mg/dL)
Yoga	181.80 ± 39.75	176.64 ± 38.59 *†	150.42 ± 70.52	153.51 ± 72.88 *†	103.54 ± 34.09	98.65 ± 33.67 *†	49.30 ± 11.48	48.61 ± 11.55 *†
Control	183.44 ± 40.33	193.27 ± 47.27 *	155.86 ± 79.40	191.12 ± 107.44 *	103.99 ± 33.00	108.01 ± 40.4 *	48.92 ± 11.53	44.62 ± 12.15 *

There were significantly better reductions in TC, TG and LDL and increases in HDL in the yoga group than the control. * Paired sample *t*-test significance *p* < 0.001. † ANCOVA *p* < 0.001. TC: total cholesterol, TG: triglycerides, LDL: low-density lipoprotein, HDL: high-density lipoprotein.

**Table 3:** Comparison of three subgroups (lesser than, within, and above normal range) of baseline lipid values in control and yoga groups of individuals with high risk for diabetes after three months of intervention. TC: Total Cholesterol; TG: Triglyceride; LDL: low-density lipoprotein; HDL: high-density lipoprotein.

Groups	Lipid Categories	Diabetes A1c (≥6.5)			Prediabetes (A1c 5.3–6.4) with Diabetes High Risk (IDRS ≥ 60)			Normoglycemia (A1c <5.3) with Diabetes High Risk (IDRS ≥60)		
		Pre, mg/dL	Post, mg/dL	Diff (%)	Pre, mg/dL	Post, mg/dL	Diff (%)	Pre, mg/dL	Post, mg/dL	Diff (%)
Yoga	TC (<150)	131.35 ± 15.76	167.62 ± 37.67	−36.27 *† (27.6%)	130.25 ± 16.20	160.94 ± 37.02	−30.69 * (23.5%)	131.80 ± 15.30	163.83 ± 39.55	−32.03 * (24.3%)
	TC (150–200)	175.32 ± 14.18	176.61 ± 38.97	−1.29 (0.73%)	174.25 ± 13.93	174.92 ± 36.88	−0.67† (0.38%)	175.61 ± 13.72	174.50 ± 35.34	1.11 (0.63%)
	TC (>200)	228.71 ± 24.20	185.83 ± 37.61	42.87 *† (18.7%)	230.24 ± 25.64	187.10 ± 39.79	43.14 *† (18.7%)	232.64 ± 27.93	187.55 ± 39.07	45.09 *(19.3%)
	TG (<150)	104.97 ± 26.33	147.60 ± 71.72	−42.62 *† (40.6%)	105.63 ± 25.70	143.61 ± 67.49	−37.98 *† (35.9%)	104.96 ± 25.49	148.03 ± 70.88	−43.07 * (41.0%)
	TG (150–200)	173.51 ± 14.58	155.25 ± 71.17	18.26 *† (10.5%)	171.58 ± 14.19	155.81 ± 61.34	15.77 *† (9.19%)	173.06 ± 14.55	169.29 ± 81.49	3.77 (2.17%)
	TG (>200)	266.78 ± 56.77	164.65 ± 74.78	102.18 *† (38.3%)	267.97 ± 60.35	173.76 ± 81.09	94.21 *† (35.1%)	262.82 ± 55.22	168.69 ± 80.60	94.13 * (35.8%)
	LDL (<100)	76.68 ± 15.96	95.04 ± 34.41	−18.36 *† (23.9%)	76.55 ± 16.76	92.66 ± 33.15	−16.11 * (21.0%)	76.56 ± 17.02	92.19 ± 31.17	−15.37 * (20.0%)
	LDL (100–130)	113.79 ± 8.53	101.32 ± 31.96	12.47 *† (10.9%)	113.06 ± 8.18	98.34 ± 31.88	14.72 *† (13.0%)	113.91 ± 8.27	100.67 ± 31.93	13.23 * (11.6%)
	LDL (>130)	152.75 ± 20.71	107.06 ± 35.31	45.68 *† (29.9%)	154.14 ± 21.15	108.14 ± 35.04	46.0 *† (29.8%)	155.41 ± 24.62	108.25 ± 35.03	47.15 * (30.3%)
	HDL (<45)	38.69 ± 5.26	47.01 ± 11.43	−8.31 *† (21.4%)	38.48 ± 5.17	45.98 ± 11.34	−7.5 (19.4%)	38.70 ± 5.24	46.34 ± 11.32	−7.64 * (19.7%)
	HDL (45–60)	52.12 ± 4.14	49.39 ± 11.54	2.72 *† (5.2%)	52.25 ± 4.28	50.12 ± 11.40	2.13 *† (4.0%)	52.07 ± 4.21	49.22 ± 11.06	2.85 * (5.4%)
	HDL (>60)	67.86 ± 6.74	51.36 ± 11.51	16.49 *† (24.3%)	67.66 ± 6.94	51.25 ± 11.56	16.41 *† (24.2%)	69.07 ± 9.11	50.84 ± 12.40	18.23 * (26.3%)
Control	TC (<150)	130.65 ± 15.57	170.51 ± 40.59	−39.86 * (30.5%)	130.40 ± 14.89	167.51 ± 37.52	−37.10 * (28.4%)	128.57 ± 14.05	180.68 ± 52.30	−52.11 * (40.5%)
	TC (150–200)	175.53 ± 14.17	182.58 ± 41.71	−7.05 * (4%)	175.82 ± 13.77	182.46 ± 40.62	−6.63 * (3.8%)	173.26 ± 14.12	181.01 ± 42.40	−7.74 * (4.46%)
	TC (>200)	228.75 ± 27.34	224.04 ± 44.16	4.70 * (2.0%)	227.27 ± 24.48	221.15 ± 40.59	6.11 * (2.7%)	229.43 ± 25.02	225.69 ± 40.98	3.74 * (1.63%)
	TG (<150)	104.76 ± 25.95	162.69 ± 102.67	−57.93 * (55.2%)	106.41 ± 26.33	174.32 ± 111.57	−67.90 * (63.8%)	106.68 ± 27.13	164.42 ± 100.67	−57.73 * (54.1%)
	TG (150–200)	173.09 ± 14.65	158.38 ± 80.19	14.71 * (3.18%)	173.60 ± 15.59	167.25 ± 86.19	6.34 * (3.6%)	177.69 ± 16.44	165.00 ± 88.74	12.69 (7.1%)
	TG (>200)	278.24 ± 64.15	287.10 ± 75.54	−8.85 * (3.1%)	274.04 ± 64.22	284.63 ± 74.75	−10.59 * (3.8%)	278.12 ± 67.55	288.13 ± 75.20	−10.01 * (4.5%)
	LDL (<100)	76.79 ± 16.08	95.78 ± 33.91	−18.98 * (24.7%)	76.71 ± 16.03	100.65 ± 36.26	−23.94 * (31.2%)	75.95 ± 16.96	98.89 ± 34.74	−22.94 * (30.2%)
	LDL (100–129)	114.44 ± 8.47	99.44 ± 34.25	14.99 * (13.0%)	113.98 ± 8.61	100.90 ± 38.79	13.08 * (11.4%)	114.78 ± 8.68	94.11 ± 34.02	20.67 * (18.0%)
	LDL (>130)	151.63 ± 18.93	146.32 ± 37.31	5.31 * (3.5%)	149.74 ± 16.28	143.78 ± 18;25	5.96 * (3.98%)	151.70 ± 18.68	145.87 ± 39.92	5.82 (3.8%)
	HDL (<45)	38.86 ± 5.00	38.21 ± 7.53	0.44 (1.1%)	38.13 ± 4.98	37.03 ± 7.13	1.10 * (2.8%)	37.90 ± 4.64	38.25 ± 6.61	−0.34 (0.89%)
	HDL (45–60)	51.96 ± 4.13	48.68 ± 12.81	3.28 * (6.3%)	51.62 ± 4.28	48.85 ± 13.44	2.76 * (5.2%)	51.67 ± 3.89	49.11 ± 13.18	2.55 * (4.9%)
	HDL (>60)	67.90 ± 6.08	51.45 ± 11.88	16.44 * (24.2%)	68.24 ± 6.21	49.75 ± 11.85	18.48 * (27.0%)	67.28 ± 5.00	50.92 ± 11.66	16.35 * (24.3%)

* Paired sample *t*-test significance *p* < 0.001, * 2 Paired sample *t*-test significance *p* = 0.041, † ANCOVA *p* < 0.001. NORMALIZING EFFECT OF YOGA there was a significant increase in those with low baseline values; non-significant changes in those in the normal range; and a reduction in those with abnormally high baseline values.

**Table 4:** Changes in lipid profile in two groups after three months in gender, area (rural/urban), and age subgroups.

Groups			TC (mg/dL)			TG (mg/dL)			LDL (mg/dL)			HDL (mg/dL)		
		Y/C	Pre	Post	Df	Pre	Post	Df	pre	Post	Df	pre	Post	df
Gender	Male	Y	181.96 ± 39.77	177.44 ± 38.43	4.52	150.02 ± 69.88	154.33 ± 73.82	−4.31	103.5 ± 34.72	98.73 ± 33.36	4.78	49.3 ± 11.53	48.90 ± 11.83	0.48
		C	181.24 ± 39.63	192.73 ± 47.73	−11.49	153.74 ± 77.62	189.80 ± 108.57	−36.06	104.88 ± 32.90	109.07 ± 41.25	−4.19	49.30 ± 11.55	44.77 ± 12.26	4.52
	Female	Y	181.68 ± 39.74	176.03 ± 38.70*	5.64	150.73 ± 71.01	152.89 ± 72.17	−2.16	103.56 ± 33.61	98.59 ± 33.90*	4.96	49.24 ± 11.44	48.39 ± 11.34	0.84
		C	184.96 ± 40.75	193.65 ± 46.95	−8.68	157.34 ± 80.60	192.04 ± 106.65	−34.69	103.37 ± 33.05	107.28 ± 39.88	−3.90	48.66 ± 11.51	44.51 ± 12.08	4.15
Location	Urban	Y	181.30 ± 39.74	176.66 ± 38.74	4.63	150.16 ± 7070.62	153.87 ± 73.05	−3.70	103.07 ± 34.11	98.54 ± 33.84 *	4.53	49.41 ± 11.39	48.66 ± 11.64	0.75
		C	183.30 ± 40.00	193.99 ± 47.13 *	−10.69	154.43 ± 79.19	188.01 ± 105.64	−33.58	104.35 ± 33.18	107.7 ± 40.14 *	−3.41	49.00 ± 11.67	44.94 ± 12.40 *	4.06
	Rural	Y	182.89 ± 39.77	176.58 ± 38.39 *	6.31	150.99 ± 70.33	152.72 ± 72.51	−1.72	104.55 ± 34.04	98.88 ± 33.31 *	5.66	49.07 ± 11.67	48.51 ± 11.37	0.56
		C	183.59 ± 40.70	192.48 ± 47.42	−8.8	157.45 ± 79.63 *	194.56 ± 109.32	−37.11	103.5 ± 32.79 *	108.2 ± 40.80 *	−4.69	48.83 ± 11.37	44.26 ± 11.87 *	4.57
Age groups	<40	Y	180.80 ± 38.78	177.0 ± 38.38 *	3.78	149.63 ± 73.17	150.10 ± 70.10	−0.46	102.28 ± 33.28	99.62 ± 33.40	2.65	49.33 ± 11.16	48.83 ± 11.21	0.50
	<40	C	182.42 ± 40.51	195.43 ± 46.12	−11.00	155.05 ± 78.79	191.03 ± 109.61	−35.98	104.28 ± 32.65	107.91 ± 40.93	−3.63	49.30 ± 11.52	44.88 ± 12.46	4.42
	>40	Y	182.18 ± 39.93	176.49 ± 38.85 *	5.68	150.71 ± 69.49	154.90 ± 73.79 *	−4.18	103.92 ± 34.33	98.28 ± 33.92 *	5.64	49.34 ± 11.54	48.55 ± 11.54 *	0.79
	>40	C	183.10 ± 40.27	192.53 ± 47.65 *	−9.43	156.14 ± 79.63	191.15 ± 106.70 *	−35.00	103.89 ± 33.12	108.0 ± 40.29 *	−4.15	48.79 ± 11.53	44.53 ± 12.05 *	4.26

*p < 0.001 paired samples *T* test (pre-post within groups). There were no significant differences between males and females, urban and rural, or young and old age groups. (ANCOVA between the two subgroups).

## Data Availability

All the associated data is available within the manuscript/Supplementary Materials.

## Supplementary Materials

The following are available online at https://www.mdpi.com/article/10.3390/medicines8070037/s1, Figure S1: Map of India showing the geographic spread of the intervention sites (reusing the figure after permission (Nagendra et al., 2019), Figure S2: Graphs showing the normalizing effect of yoga, Table S1: Validated yoga lifestyle protocol for prediabetes and uncomplicated diabetes.

## Abbreviations

DYP	Diabetes yoga lifestyle protocol
NMB	Niyantrita Madhumeha Bharata Abhiyaan
NABL	National Accreditation Board for Testing and Calibration Laboratories
A1c	Glycated hemoglobin
TC	Total cholesterol
TG	Triglyceride
LDL	Low-density lipoprotein
HDL	High-density lipoprotein
VLDL	Very-low-density lipoprotein
T2DM	Type-2 diabetes mellitus
CHD	Coronary heart disease
ADA	American Diabetes Association
ANS	Autonomic nervous system
SAM	Sympatho-adrenal medullary
HPA	Hypothalamic pituitary adrenal
NO	Nitric oxide
IDRS	Indian diabetes risk score
IYA	Indian Yoga Association
CEB	Census enumeration blocks
SRF	Senior research fellows
ASHA	Accredited social health activists
IEC	Institutional ethics committee
