# Supplementary material for: Effectiveness of Yoga Lifestyle on Lipid Metabolism in a Vulnerable Population—A Community Based Multicenter Randomized Controlled Trial
Source: Medicines (Basel). 2021 Jul 13;8(7):37. doi: 10.3390/medicines8070037 (PMC8303653; doi:10.3390/medicines8070037)
Supplement: Supplementary file 1 [file medicines-08-00037-s001.zip › medicines-1188149-supplementary.pdf]

Supplementary Materials: Effectiveness of Yoga Lifestyle on Lipid Metabolism in a Vulnerable Population—A Community Based Multicenter Randomized Controlled Trial

Raghuram Nagarathna, Saurabh Kumar, Akshay Anand, Ishwara N. Acharya, Amit Kumar Singh, Suchitra S. Patil, Ramesh H Latha, Purnima Datey and Hongasandra Ramarao Nagendra

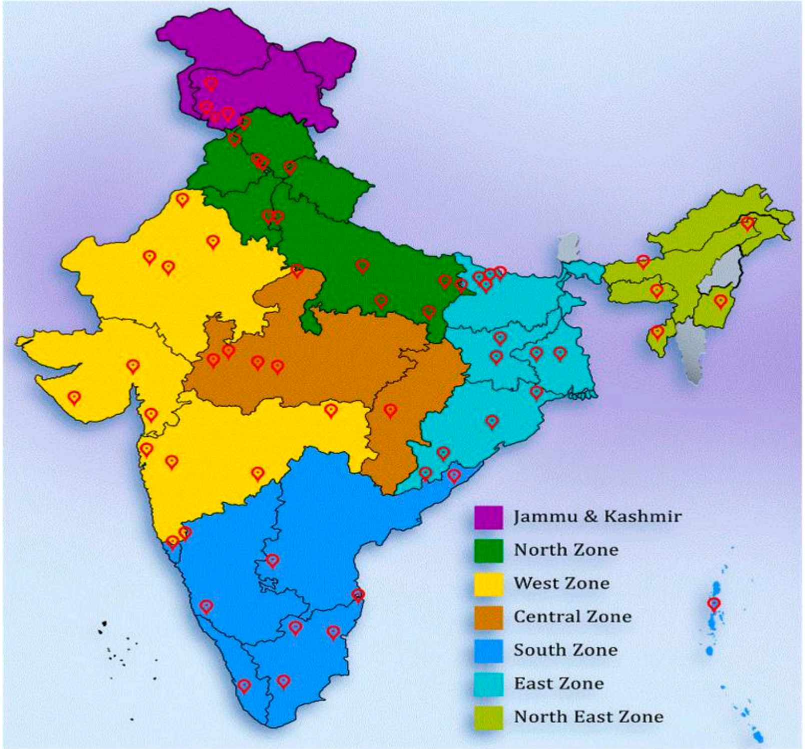

Figure S1. Map of India showing the geographic spread of the intervention sites (reusing the figure after permission (Nagendra et al., 2019).

Supplementary Figure S2: Graphs showing the normalizing effect of yoga:

Triglycerides (mg/dL) yoga (n-3983)	Triglycerides (mg/dL) control (n-4183.)
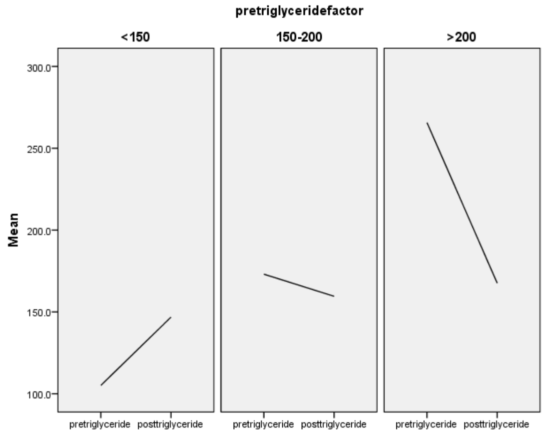	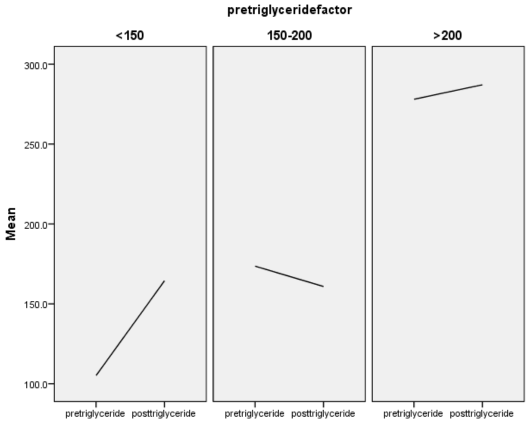

significant increase in those with Tg< normal, decrease in those with TG>normal, no change in normal range. Non-significant changes in control group

LDL mg/dl yoga (n=3983)	LDL mg/dl control (n=4183)
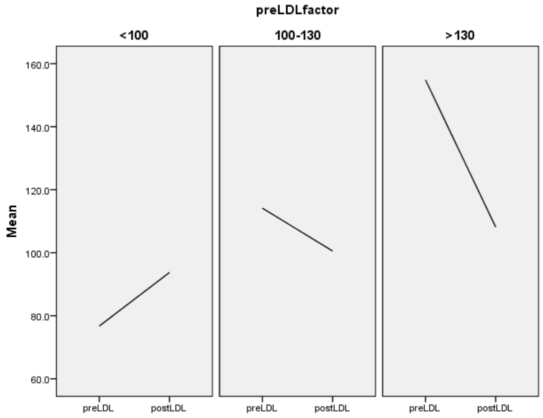	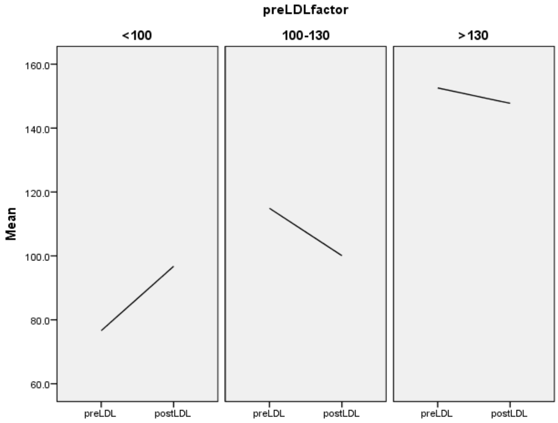

Significant increase in those with LDL< normal, decrease in those with LDL>normal, no change in normal range. Non-sigbificant similar changes in control group

Figure S2. Graphs showing the normalizing effect of yoga.

Table S1. Validated diabetes yoga lifestyle protocol for prediabetes and uncomplicated diabetes.

**Table S1.** Validated yoga lifestyle protocol for prediabetes and uncomplicated diabetes			
**S. No**	**Name of Practice**	**Time (min)**	**Benefits**
1	Opening Prayer: *Asatoma Sad gamaya* *Tamaso ma Jyotir - gamaya* *Mrtyor-Maa Amrtamgamaya* *Om Shaantih Shaantih Shaantih* Meaning: From ignorance lead me to truth; from darkness, lead me to light; from death, lead me to immortality; Om peace, peace, peace.	2	Enhances the benefits of practice

2	Loosening Exercises (preparatory *Sukshma Vyayamas* and *Shithilikarna* practices)<br>*Urdhava Hastashvasan* (upward tree position)<br>*(hand stretching breathing 3 rounds at 90º, 135º and 180º each)*<br>*Kati-Shakti Vikasaka* (3 rounds)<br>*a). Forward and Backward Bending*<br>*b). Twisting*<br>Sarvanga Pushti (Rotatory circular movements)<br>(3 rounds clockwise, 3 rounds anticlockwise)	6	Warms up the body
3	*Surya Namaskara* (SN) (sun salutation)<br>10 step fast *Surya Namaskara* (fast sun salutation) 6 rounds<br>12 step slow *Surya Namaskara* (slow sun salutation 1 round<br>Modified version Chair SN 7 rounds	9	reinforces the complete system/body
4	*Asanas* (body pose/posture) (1min per *asana*)<br>*1.Standing Position* (1min per asana)<br>*Trikonasana* (extended triangle pose),<br>*Parvritta Trikonasana* (revolved triangle pose),<br>*Prasarita Padhastasana* (wide-legged forward bend)<br>*2. Supine Position*<br>*Jatara Parivartanasana* (master revolved abdomen pose)<br>*Pawanmuktasana* (wind-relieving pose),<br>*Viparitakarani* (upside-down pose)<br>*3.Prone Position*<br>*Bhujangasana* (cobra pose),<br>*Dhanurasana* (bow pose),<br>*4.Sitting Position*<br>*Mandukasana* (frog pose),<br>*Vakrasana/Ardhamatsayendrasan*a (half spinal twist pose),<br>*Paschimatanasana* (seated forward bend),<br>*Ardha Ushtrasana* (half camel pose)<br>At the end, relaxation with abdominal breathing in supine position (*vishranti*), 10-15 rounds (2 minutes)	15	brings peace to the mind and body, also prepares the body for deeper spiritual practices
5	*Kriya* (outward physical manifestation)<br>*Agnisara (*abdomen churning*)*:1 minute<br>*Kapalabhati* (forehead Shining Breathing Technique) *(@60 breaths per minute for 1 minute followed by rest for 1 minute)*	3	increases the internal energy, and improves circulation respectively
6	*Pranayama* (breathing techniques)<br>*Nadishuddhi (*alternate nostril breathing*) (for 6 minutes, with antarkumbhak* (internal breath retention*) and Jalandhar bandh (*chin lock*) (for 2 seconds)*<br>*Bhramari* (humming bee breathing) *3 minutes*	9	acts as scavenger by cleaning the nadis/channels
7	Meditation *(for Stress, for deep relaxation and silencing of mind)*<br>Cyclic Meditation	15	concentration, relaxation and silencing of the mind
8	Resolve *(I am completely healthy)*		
9	Closing Prayer: *Sarve Bhavantu Sukhina*<br>*Sarve Santu Niramayaah*<br>*Sarve Bhadrani Paśyantu*	1	Instills feeling of human brotherhood

Maa Kashcid-Duhkha-Bhag-Bhavet<br>*Om Shaantih Shaantih Shaantih*<br>Meaning: Let all be happy, free from diseases. Let all align with reality, let no-one suffer from miseries. Om peace, peace, peace.	
Total duration	60 mins
